# Expression-based clustering of CAZyme-encoding genes of *Aspergillus niger*

**DOI:** 10.1186/s12864-017-4164-x

**Published:** 2017-11-23

**Authors:** Birgit S. Gruben, Miia R. Mäkelä, Joanna E. Kowalczyk, Miaomiao Zhou, Isabelle Benoit-Gelber, Ronald P. De Vries

**Affiliations:** 1 Fungal Physiology, Westerdijk Fungal Biodiversity Institute, Uppsalalaan 8, 3584 CT Utrecht, The Netherlands; 2 Microbiology, Utrecht University, Padualaan 8, 3584 CH Utrecht, The Netherlands; 3 Fungal Molecular Physiology, Utrecht University, Uppsalalaan 8, 3584 CT Utrecht, The Netherlands; 4 Department of Food and Environmental Sciences, Division of Microbiology and Biotechnology, Viikki Biocenter 1, University of Helsinki, Helsinki, Finland; 5 Current affiliation: ATGM, Avans University of Applied Sciences, Lovensdijkstraat 61–63, 4818 AJ Breda, The Netherlands; 6 Current affiliation: Center for Structural and Functional Genomics, Concordia University, 7141 Sherbrooke St. W, Montreal, QC Canada

**Keywords:** Transcriptional regulators, Plant biomass degradation, CAZy genes, XlnR, AmyR, GalX, AraR, RhaR, *Aspergillus niger*

## Abstract

**Background:**

The *Aspergillus niger* genome contains a large repertoire of genes encoding carbohydrate active enzymes (CAZymes) that are targeted to plant polysaccharide degradation enabling *A. niger* to grow on a wide range of plant biomass substrates. Which genes need to be activated in certain environmental conditions depends on the composition of the available substrate. Previous studies have demonstrated the involvement of a number of transcriptional regulators in plant biomass degradation and have identified sets of target genes for each regulator. In this study, a broad transcriptional analysis was performed of the *A. niger* genes encoding (putative) plant polysaccharide degrading enzymes. Microarray data focusing on the initial response of *A. niger* to the presence of plant biomass related carbon sources were analyzed of a wild-type strain N402 that was grown on a large range of carbon sources and of the regulatory mutant strains Δ*xlnR*, Δ*araR*, Δ*amyR*, Δ*rhaR* and Δ*galX* that were grown on their specific inducing compounds.

**Results:**

The cluster analysis of the expression data revealed several groups of co-regulated genes, which goes beyond the traditionally described co-regulated gene sets. Additional putative target genes of the selected regulators were identified, based on their expression profile. Notably, in several cases the expression profile puts questions on the function assignment of uncharacterized genes that was based on homology searches, highlighting the need for more extensive biochemical studies into the substrate specificity of enzymes encoded by these non-characterized genes. The data also revealed sets of genes that were upregulated in the regulatory mutants, suggesting interaction between the regulatory systems and a therefore even more complex overall regulatory network than has been reported so far.

**Conclusions:**

Expression profiling on a large number of substrates provides better insight in the complex regulatory systems that drive the conversion of plant biomass by fungi. In addition, the data provides additional evidence in favor of and against the similarity-based functions assigned to uncharacterized genes.

**Electronic supplementary material:**

The online version of this article (10.1186/s12864-017-4164-x) contains supplementary material, which is available to authorized users.

## Background

*Aspergillus niger* is a saprobic fungus that degrades a broad range of plant polysaccharides. Its genome encodes a versatile set of polysaccharide degrading enzymes [1, 2], which can be classified into families of glycoside hydrolases (GHs), polysaccharide lyases (PLs), carbohydrate esterases (CEs) and auxiliary activities (AAs) according to the CAZy (Carbohydrate-Active Enzymes) database (www.cazy.org; [3]). The classification is based on amino acid sequence and structural similarity. Among the 176 genes of *A. niger* CBS513.88 [4] that are predicted to encode CAZymes involved in plant biomass degradation less than half have been biochemically characterized, while the others have been assigned to CAZy families merely based on homology to functionally characterized genes.

In addition to the production of a wide variety of CAZyme encoding genes, the efficient depolymerization of the polysaccharides present in plant biomass requires a fine-tuned regulatory system. The expression of fungal CAZy genes have been shown to be controlled by multiple transcriptional regulators, most of which belong to fungi specific Zn_2_Cys_6_zinc binuclear family of transcriptional factors [5]. In *A. niger*, several regulators related to plant polysaccharide degradation have been identified [6]. These include XlnR [7], AraR [1], AmyR [8], InuR [9], RhaR [10], ManR/ClrB [11, 12], ClrA [13], GalX [14] and GaaR [15] that have been reported as transcriptional activators of CAZymes (Table 1). These regulators respond to mono- and small oligosaccharides that act as inducers (Table 1) [16], but so far, a limited set of target genes of these regulators have been identified. While some genes can be controlled by a single regulator, co-regulation of several CAZyme encoding genes has been described in *Aspergillus* species.

**Table 1:** Transcriptional activators involved in plant polysaccharide degradation and/or sugar catabolism in *A. niger*

Regulator^a^	Inducer	Function	Reference
AmyR	D-glucose or maltose	Starch degradation	[8]
AraR	L-arabitol	Arabinan degradation, Pentose Catabolic Pathway	[22, 66]
ClrA	cellulose^b^	Degradation of cellulose	[13]
GaaR	2-keto-3-deoxy-L-galactonate	Degradation of polygalacturonic acid and more complex pectins, transport of D-galacturonic acid, D-galacturonic acid catabolism	[29, 47]
GalX	D-galactose or derivative	D-galactose catabolism	[15]
InuR	sucrose	Inulin and sucrose degradation	[9, 43]
ManR/ClrB		Galactomannan and cellulose degradation	[11, 12]
RhaR	L-rhamnose derivative	Rhamnogalacturonan I degradation, L-rhamnose catabolism	[10, 27]
XlnR	D-xylose	Xylan, xyloglucan and cellulose degradation, Pentose Catabolic Pathway	[7, 21, 22]

^a^The *A. niger* deletion strains of the underlined regulators were used in this study

^b^Based on data from *Neurospora crassa* [45]

AmyR, a transcriptional regulator that controls the genes involved in starch degradation, was the first well-studied regulator in several *Aspergillus* species [17, 18]. In Aspergilli, AmyR is induced by maltose and regulates genes encoding α-amylases, glucoamylase and α-glucosidases all of which are involved in depolymerization of starch, the major storage polysaccharide in plants [6]. In addition, AmyR has been shown to have a broader physiological role in *A. niger* by controlling some of the genes encoding D-glucose and D-galactose releasing enzymes, i.e. β-glucosidases, and α- and β-galactosidases [8]. Also, D-glucose or its metabolic product has been suggested to have a possible role as the inducer of the AmyR system in *A. niger*.

XlnR has an important role in biomass degradation by controlling the expression of genes encoding enzymes that degrade xylan, cellulose and xyloglucan, which are the most abundant polysaccharides in nature [19–21]. The *xlnR* gene has also been shown to be present in almost in all filamentous ascomycete fungi [22]. The range of genes regulated by XlnR include genes encoding endoxylanase, β-xylosidase, α-glucuronidase, acetylxylan esterase, arabinoxylan arabinofuranohydrolase, feruloyl esterase, α- and β-galactosidases, endoglucanase and cellobiohydrolase, as well as *aglB* and *lacA* genes that encode enzymes putatively involved in xyloglucan or galactomannan degradation [23].

A homolog of XlnR, AraR, is a transcriptional regulator induced by L-arabinose and its degradation product, L-arabitol [22]. These monomers are building blocks of arabinan present in side chains of arabinoxylan and pectin. Two arabinan hydrolyzing enzymes produced by *A. niger*, α-L-arabinofuranohydrolases A and B, are controlled by AraR [22]. In addition, AraR controls the expression of the genes involved in L-arabinose catabolism. AraR and XlnR also co-regulate genes from pentose catabolic pathway and pentose phosphate pathway [24].

The expression of the genes encoding inulinases and invertase, which hydrolyze plant storage polymer inulin, is controlled by the transcriptional regulator InuR in *A. niger* [9]. Inulinolytic enzyme encoding genes are also inducted by sucrose, and moreover, the repertoire of the genes regulated by InuR has been suggested to include other genes related to degradation of inulin and sucrose.

Several plant polysaccharides, e.g. xylan, pectin and glucogalactomannan, include D-galactose, which is released by fungal α- and β-galactosidases and endogalactanases. While a galactose-related regulator GalR was reported to be unique for *Aspergillus nidulans* [25], it has also been found in related species of *Aspergillus* section *nidulantes* [26]. In contrast, GalX is more generally present in *Aspergillus* species. In *A. niger*, GalX regulates the expression of the genes from oxido-reductive pathway for D-galactose catabolism [14].

In addition to AraR, the other pectinolytic regulators described from *A. niger* are RhaR [10] and GaaR [15]. RhaR, induced by a metabolic conversion product of L-rhamnose, influences the degradation of rhamnogalacturonan I part of pectin by controlling several genes involved in L-rhamnose release and catabolism [10, 27], as well as a L-rhamnose transporter [28]. The more recently described transcriptional regulator GaaR is induced by 2-keto-3-deoxy-L-galactonate, a metabolic conversion product of D-galacturonic acid, and involved in the release of galacturonic acid from polygalacturonic acid and more complex pectins, as well as transport of galacturonic acid and induction of the galacturonic acid catabolic genes [15, 29].

Other plant-biomass related transcriptional regulators described in *Aspergillus* species include the regulator of mannan degrading enzymes, ManR/ClrB, which was first described in *Aspergillus oryzae*, with a role in galactomannan and cellulose degradation [11, 12]. In *A. oryzae,* ManR/ClrB is induced by the disaccharide mannobiose, but not mannose [11, 12]. Furthermore, the genomes of Aspergilli possess various homologs of plant-polysaccharide related regulators from other fungal species, and the gene expression studies have also indicated the presence of several additional regulators involved in this process, including e.g. those responding to the presence of ferulic acid, glucuronic acid and galacturonic acid [6].

The aim of this study was to evaluate co-regulation/co-expression of characterized and putative CAZymes to gain more insight in the function of uncharacterized CAZyme encoding genes in plant biomass utilization and to identify new targets of transcriptional regulators. The focus of the study was on the initial response of *A. niger* to the presence of a carbon source. For this, microarray data were analyzed of *A. niger* N402 (wild type) that was grown on a set of 23 carbon sources (including eight monosaccharides, two oligosaccharides, 11 polysaccharides, a crude plant biomass substrate and ferulic acid), and of regulatory mutant strains (Δ*xlnR*, Δ*araR*, Δ*amyR*, Δ*rhaR* and Δ*galX*) that were grown on their specific inducing compounds. Hierarchical clustering of the expression data revealed several gene clusters that appear to be under control of the same regulators.

## Results and discussion

Microarray data were analyzed of *A. niger* N402 that was grown on 23 carbon sources (Tables 2, 3) and of the regulatory mutants Δ*xlnR*, Δ*araR*, Δ*amyR*, Δ*rhaR* and Δ*galX* that were grown on their inducing compounds (Tables 1, 3). The mycelial samples were collected after 2 h or 4 h (for N402 and Δ*amyR* on D-maltose) exposure to the carbon source of interest. Thus, this study focused on the initial response to the presence of a carbon source to avoid changes due to carbon source limitation or changes in the polymeric substrates. Although we can therefore not exclude that genes that were not expressed may have been induced after longer incubation times or on substrates that were not included in this analysis, it provides a detailed understanding of the initial response of *A. niger* to the presence of plant-biomass related carbon sources.

**Table 2:** *A. niger* strains used in this study

Strain	Genotype	Reference
*A. niger* N402	*cspA1*	[67]
FP-304 (Δ*rhaR*)	*cspA1*, Δ*kusA*::*amdS*+, *pyrA5*, Δ*rhaR*::*pyrA*+	[10]
UU-A101.1 (Δ*amyR*)	*cspA1*, Δ*argB*, *pyrA6*, *leuA1*, *nicA1*, Δ*amyR*::*argB*+	[8]
FP-306 (Δ*galX)*	*cspA1*, Δ*kusA*::*amdS*+, *pyrA5*, Δ*galX*::*pyrA*+	[14]
UU-A033.21 (Δ*araR)*	*cspA1*, *pyrA6*, *nicA1*, *leuA1*, Δ*argB*::pIM2101 Δ*araR*::*argB*+	[22]
UU-A062.10 (Δ*xlnR)*	*cspA1*, *pyrA6*, Δ*argB*, *nicA1*, *leuA*, *pyrA6*, Δ*xlnR*::*pyrA*+	[22]

**Table 3:** Composition, purity and concentration of the carbon sources used in this study

Substrate	Conc.	Company	Purity	Composition
D-glucose	25 mM	Sigma-Aldrich	≥99.5%	–
D-fructose	25 mM	Sigma-Aldrich	≥99%	–
D-galactose	25 mM	Sigma-Aldrich	≥99%	–
L-arabinose	25 mM	Sigma-Aldrich	≥99%	–
D-xylose	25 mM	Sigma-Aldrich	≥99%	–
D-mannose	25 mM	Sigma-Aldrich	≥99%	–
D-galacturonic acid	25 mM	Sigma-Aldrich	≥99%	–
L-rhamnose	25 mM	Sigma-Aldrich	≥99%	–
Maltose	25 mM	Sigma-Aldrich	≥99%	–
Sucrose	25 mM	Sigma-Aldrich	≥99%	–
Ferulic acid	25 mM	Sigma-Aldrich	≥99%	–
Inulin from chicory	1%	Sigma-Aldrich	ns	ns
Cellulose	1%	Sigma-Aldrich	ns	ns
Guar gum	1%	Sigma-Aldrich	ns	ns
Xyloglucan	1%	Sigma-Aldrich	ns	ns
Xylan (from beechwood)	1%	Sigma-Aldrich	>90%	>90% D-xylose residues
Polygalacturonic acid (from apples)	1%	Sigma-Aldrich	≥85%	ns
Apple pectin	1%	Sigma-Aldrich	ns	ns
Galactan (from potato)	1%	Megazyme	ns	Gal: Ara: Rha: GalA = 88: 2: 3: 7
Debranched 1,5-α-L-arabinan (from sugar beet)	1%	Megazyme	~95%	Ara: Gal: Rha: GalA = 88: 4: 2: 6
Rhamnogalacturonan I (from potato)	1%	Megazyme	>97%	GalA: Rha: Ara: Xyl: Gal: Os = 62: 20: 3.3: 1: 12: 1.7
Mannan (ivory nut)	1%	Megazyme	>98%	99% Mannan, Ara + Xyl traces
Citrus pulp	1%	ns	ns	Glc: GalA: Ara: Gal: Xyl: Man: Rha = 39: 35: 11: 7: 4: 4: 1
Sugar beet pulp	1%	ns	ns	Glc: Ara: GalA: Gal: Xyl: Man: Rha = 33: 28: 26: 7: 2: 2: 1
Soy bean hulls	1%	ns	ns	Glc: GalA: Xyl: Ara: Man: Gal: Rha = 49: 16: 15: 8: 7: 4: 1

*Gal* D-galactose, *Ara* L-arabinose, *Rha* L-rhamnose, *GalA* D-galacturonic acid, *Xyl* D-xylose, *Man* D-mannose, *Os* other sugars. ns = not specified

Clustering the expression profiles of *A. niger* (putative) CAZyme encoding genes that are related to plant polysaccharide degradation by Pearson correlation resulted in nine clusters, A-I (Additional file 1). After the initial clustering analysis, genes with a signal value below 50 under all growth conditions were removed from the analysis. These genes were considered not to be significantly expressed (Additional files 2 and 3). The genes that were significantly expressed (signal value >50) at least under one condition are shown in Tables 4, 5, 6, 7, 8, 9, 10, 11. In addition, the fold-changes of the significantly expressed genes between N402 and the regulatory mutant strains were determined (Tables 4, 5, 6, 7, 8, 9, 10, 11). Negative fold-changes indicate genes for which the expression is lower in the mutant than in the wild type strain, while positive fold-changes indicate higher expression in the mutant than in the wild type strain. If the negative fold-change is larger than 2.5, we consider this gene under control of the respective regulator.

**Table 4:** Significantly expressed *A. niger* N402 genes from cluster B in the expression profiling tree

				Up/Down-regulated^c^					Regulated by		
	Gene^a^	Enzyme^b^	CAZy family	Δ*xlnR*	Δ*araR*	Δ*rhaR*	Δ*galX*	Δ*amyR*	This study	Literature	Reference
B-1	An03g01050	GLN	GH5	4.8	**−74.6**	3.1	29.9	6.3	AraR	nr	[38]
	An14g01800 (*aglD*)	AGL	GH27	−1.5	**−97.4**	–	–	–	AraR	nr	[38]
	An01g00330 (*abfA*)	ABF	GH51	1.0	**−113.9**	−1.7	4.6	38.3	AraR	AraR, GaaR	[15, 22, 34, 68]
	An03g00940 (*xlnC*/*xynA*)	XLN	GH10	**−17.6**	**−27.6**	–	–	–	AraR, XlnR	ClrA, XlnR	[13, 21, 38]
	An03g00960 (*axhA*)	AXH	GH62	**−17.3**	**−28.5**	–	–	–	AraR, XlnR	AraR, XlnR	[21, 22, 30]
	An16g06800 (*eglB*)	EGL	GH5	4.1	**−6.2**	−1.3	−1.1	1.0	AraR	nr	[38]
	An15g04550 (*xynA*)	XLN	GH11	–	**−6.6**	–	–	–	AraR	nr	[38]
B-2	An12g05010 (*axeA*)	AXE	CE1	**−51.5**	1.5	–	–	–	XlnR	XlnR	[21, 38]
	An01g09960 (*xlnD*/*xynD*)	BXL	GH3	**−502.6**	1.6	−1.8	2.1	5.9	XlnR	XlnR	[21, 69]
	An08g01900	BXL	GH43	**−4.0**	1.2	–	–	–	XlnR	nr	[38]
	An11g02100	BGL	GH1	**−5.3**	2.0	1.0	2.1	2.0	XlnR	nr	[38]
	An14g05800 (*aguA*)	AGU	GH67	**−32.5**	2.1	**−3.7**	1.5	1.5	RhaR, XlnR	XlnR	[21, 70]
	An09g00120 (*faeA*)	FAE	SF7^d^	**−78.3**	−1.5	–	–	–	XlnR	XlnR	[21, 36]
	An08g01760	CBH	GH6	**−3.8**	–	–	–	–	XlnR	nr	[38]
	An09g03300 (*axlA*/*xylS*)	AXL	GH31	**−10.4**	**−6.3**	1.3	3.8	4.3	AraR, XlnR	nr	[41]
	An01g00780 (*xlnB*/*xynB*)	XLN	GH11	**−40.7**	**−5.6**	1.7	1.1	−2.2	AraR, XlnR	XlnR	[21, 38]

^a^Genes with expression value of <50 in all studied *A. niger* N402 cultures are not included in the table

^b^Enzyme codes are provided in Additional file 2

^c^Fold-change between *A. niger* N402 and the regulatory mutants grown on their relevant carbon source. - = expression value <50 in both N402 and regulatory mutant strain. Negative fold-changes >2.5 were considered as proof of regulator function and are depicted in bold

^d^Sub-family (SF) classification of fungal FAEs according to Dilokpimol et al. [49]

*nr* not reported

**Table 5:** Significantly expressed *A. niger* N402 genes from cluster C in the expression profiling tree

				Up/Down-regulated^c^					Regulated by		
	Gene^a^	Enzyme^b^	CAZy family	Δ*xlnR*	Δ*araR*	Δ*rhaR*	Δ*galX*	Δ*amyR*	This study	Literature	Reference
C-1	An01g13610 (*amyD*)	AMY	GH13	1.2	–	–	1.3	–	nd	nr	[43]
	An18g04810 (*rgxC*)	RGX	GH28	–	–	**−17.1**	–	–	RhaR	GaaR, RhaR	[15, 34, 40]
	An06g00290 (*lacC*)	LAC	GH35	1.3	−1.7	**−20.2**	1.6	1.5	RhaR	AmyR, AraR, GaaR, RhaR	[15, 34, 40]
C-2	An11g04040 (*pgxA*)	PGX	GH28	–	–	–	–	–	nd	GaaR	[15, 34, 40]
	An03g06740 (*pgxB*)	PGX	GH28	–	–	–	–	–	nd	GaaR	[15, 34, 40]
	An02g12450 (*pgxC*)	PGX	GH28	−1.3	**−3.2**	–	–	–	RhaR	AraR, GaaR	[15, 34, 40]
	An12g07500 (*pgaX*)	PGX	GH28	–	–	–	–	–	nd	GaaR	[15, 34, 40]
	An02g00140 (*xynB*)	BXL	GH43	–	2.3	–	1.6	1.9	nd	GaaR	[34, 38]
	An03g06310 (*pmeA*)	PME	CE8	–	–	–	–	–	nd	GaaR, AraR, RhaR	[15, 34, 71]
	An14g04370 (*pelA*)	PEL	PL1	1.2	−1.2	−1.5	1.0	1.0	nd	GaaR	[15, 34, 72, 73]
	An14g01130 (*rglA*)	RGL	PL4	–	–	–	–	–	nd	GaaR	[15, 34, 35]
C-3	An18g05620 (*agdF*)	AGD	GH31	–	–	**−3.4**	2.7	3.2	RhaR	nr	[41]
	An09g02160 (*rgaeA*)	RGAE	CE12	–	–	**−22.1**	2.3	–	RhaR	GaaR, RhaR	[10, 34, 74]
	An04g09360 (*rgaeB*)	RGAE	CE12	–	1.5	**−38.1**	2.0	–	RhaR	GaaR, RhaR	[10, 34, 39]
	An01g14650 (*rgxA*)	RGX	GH28	–	–	**−26.4**	–	–	RhaR	RhaR	[10, 40]
	An01g06620	RHA	GH78	−1.2	1.3	**−90.2**	−1.1	2.8	RhaR	RhaR	[10, 38]
	An12g05700	RHA	GH78	–	–	**−132.0**	–	4.8	RhaR	GaaR, RhaR	[10, 34, 38]
	An14g02920 (*urhgA*)	URH	GH105	–	2.3	**−50.2**	2.1	3.6	RhaR	AraR, GaaR, RhaR	[10, 34, 39]
	An07g00240	RHA	GH78	2.0	–	**−17.0**	–	–	RhaR	RhaR	[10, 38]
	An10g00290	RHA	GH78	–	–	**−8.2**	–	–	RhaR	RhaR	[10, 38]
	An01g14600	XLN	GH11	–	–	**−13.5**	–	–	RhaR	nr	[38]
	An03g02080 (*rgxB*)	RGX	GH28	–	–	**−137.7**	–	–	RhaR	RhaR	[40]
	An04g09070	RHA	GH78	**−2.8**	1.1	**−40.4**	1.1	2.0	RhaR, XlnR	RhaR	[10, 38]

^a^Genes with expression value of <50 in all studied *A. niger* N402 cultures are not included in the table

^b^Enzyme codes are provided in Additional file 2

^c^Fold-change between *A. niger* N402 and the regulatory mutants grown on their relevant carbon source. - = expression value <50 in both N402 and regulatory mutant strain. Negative fold-changes >2.5 were considered as proof of regulator function and are depicted in bold

*nd* not detected, *nr* not reported

**Table 6:** Significantly expressed *A. niger* N402 genes from cluster D in the expression profiling tree

				Up/Down-regulated^c^					Regulated by		
	Gene^a^	Enzyme^b^	CAZy family	Δ*xlnR*	Δ*araR*	Δ*rhaR*	Δ*galX*	Δ*amyR*	This study	Literature	Reference
D-1	An02g01400 (*abnB*)	ABN	GH43	−1.6	1.4	1.6	−1.1	–	nd	nr	[39]
	An11g07660 (*exgD*)	EXG	GH5	−1.2	1.0	1.2	−1.3	−1.3	nd	nr	[38]
	An04g09890 (*agsA*)	AGS	GH13	1.4	–	–	−2.1	−1.9	nd	nr	[41, 75]
	An18g04800	RHA	GH78	–	–	1.7	1.9	–	nd	GaaR	[34, 38]
	An15g01890	BGL	GH3	1.4	1.4	1.0	−1.2	1.2	nd	nr	[38]
	An13g02110	AFC	GH29	–	–	–	–	–	nd	nr	[38]
	An01g01340	UGH	GH88	–	–	–	–	–	nd	AraR	[34, 38]
	An14g05340 (*urhgB*)	URH	GH105	–	–	–	–	–	nd	GaaR, RhaR	[34, 39]
D-2	An04g06930 (*amyC*)	AMY	GH13	–	1.0	1.2	6.2	**−3.4**	AmyR	AmyR	[38]
	An03g06550 (*glaA*)	GLA	GH15	−2.3	**−4.6**	5.9	9.1	**−44.4**	AmyR, AraR	AmyR	[8, 38, 76, 77]
	An11g03340 (*aamA*)	AMY	GH13	4.3	**−9.2**	12.8	38.4	**−35.8**	AmyR	AmyR	[41, 42]
	An04g06920 (*agdA*)	AGD	GH31	−1.1	−1.2	4.3	6.7	**−42.7**	AmyR	AmyR	[8, 38, 78]
	An12g02460 (*agtB*)	AGT	GH13	–	–	–	–	**−3.8**	AmyR	nr	[41]
	An03g05530	XG-EGL	GH12	–	–	−1.8	−1.9	**−3.0**	AmyR	nr	[4]

^a^Genes with expression value of <50 in all studied *A. niger* N402 cultures are not included in the table

^b^Enzyme codes are provided in Additional file 2

^c^Fold-change between *A. niger* N402 and the regulatory mutants grown on their relevant carbon source. - = expression value <50 in both N402 and regulatory mutant strain. Negative fold-changes >2.5 were considered proof of regulator function and are depicted in bold

nd = not detected; nr = not reported

**Table 7:** Significantly expressed *A. niger* N402 genes from cluster E in the expression profiling tree

				Up/Down-regulated^c^					Regulated by		
	Gene^a^	Enzyme^b^	CAZy family	Δ*xlnR*	Δ*araR*	Δ*rhaR*	Δ*galX*	Δ*amyR*	This study	Literature	Reference
E-1	An15g04900 (*eglD*)	LPMO	AA9	2.1	−1.2	−1.7	−1.6	−1.8	nd	nr	[38]
	An03g00190 (*pelB*)	PEL	PL1	–	–	–	1.2	1.0	nd	nr	[73]
E-2	An07g09760	BGL	GH3	–	–	–	–	–	nd	nr	[38]
	An08g01100	EXG	GH5	1.6	1.1	1.1	–	3.8	nd	nr	[38]
	An11g03200 (*inuA*/*inuB*)	INU	GH32	–	–	–	–	–	nd	InuR	[8, 79]
	An12g08280 (*inuE*/*inu1*)	INX	GH32	2.9	2.0	1.9	5.2	4.1	nd	InuR	[8]
	An02g04900 (*pgaB*)	PGA	GH28	2.6	−2.3	–	2.2	−2.1	nd	GaaR	[15, 34, 80]
	An08g11070 (*sucA*/*suc1*)	SUC	GH32	–	–	–	–	–	nd	InuR	[9, 81, 82]

^a^Genes with expression value of <50 in all studied *A. niger* N402 cultures are not included in the table

^b^Enzyme codes are provided in Additional file 2

^c^Fold-change between *A. niger* N402 and the regulatory mutants grown on their relevant carbon source. - = expression value <50 in both N402 and regulatory mutant strain

*nd* not detected, *nr* not reported

**Table 8:** Significantly expressed *A. niger* N402 genes from cluster F in the expression profiling tree

				Up/Down-regulated^c^					Regulated by		
	Gene^a^	Enzyme^b^	CAZy family	Δ*xlnR*	Δ*araR*	Δ*rhaR*	Δ*galX*	Δ*amyR*	This study	Literature	Reference
F-1	An02g00610	GUS	GH2	11.7	–	–	–	–	nd	nr	[38]
	An09g03070 (*agsE*)	AGS	GH13	1.6	1.1	−2.0	**−11.0**	**−5.8**	AmyR, GalX	nr	[43, 75]
	An16g02730 (*abnD*)	ABN	GH43	3.6	–	–	–	**−2.5**	AmyR	GaaR, RhaR	[15, 34, 39]
	An09g00260/An09g00270 (*aglC*)	AGL	GH36	1.3	1.0	−**3.5**	1.3	1.3	RhaR	AmyR	[8, 44]
F-2	An09g05350	FAE	SF9^d^	1.5	–	–	–	−1.1	nd	nr	[4]

^a^Genes with expression value of <50 in all studied *A. niger* N402 cultures are not included in the table

^b^Enzyme codes are provided in Additional file 2

^c^Fold-change between *A. niger* N402 and the regulatory mutants grown on their relevant carbon source. - = expression value <50 in both N402 and regulatory mutant strain. Negative fold-changes >2.5 were considered proof of regulator function and are depicted in bold

^d^Sub-family (SF) classification of fungal FAEs according to Dilokpimol et al. [49]

*nd* not detected, *nr* not reported

**Table 9:** Significantly expressed *A. niger* N402 genes from cluster G in the expression profiling tree

				Up/Down-regulated^c^					Regulated by		
	Gene^a^	Enzyme^b^	CAZy family	Δ*xlnR*	Δ*araR*	Δ*rhaR*	Δ*galX*	Δ*amyR*	This study	Literature	Reference
G-1	An17g00520	BGL	GH3	–	1.6	–	–	1.5	nd	nr	[38]
	An11g06540 (*mndA*)	MND	GH2	4.1	−**3.0**	**−2.8**	1.2	2.6	AraR, RhaR	nr	[4]
	An03g03740 (*blg4*)	BGL	GH1	4.1	1.0	−1.2	5.8	9.6	nd	nr	[38]
	An12g01850 (*mndB*)	MND	GH2	**−2.5**	−1.5	1.0	1.9	13.4	XlnR	nr	[38]
	An02g07590	na	GH3	–	1.6	1.0	1.3	–	nd	nr	[38]
	An09g05880 (*agdE*)	AGD	GH31	1.0	1.2	1.6	1.3	−1.1	nd	nr	[41]
G-2	An09g01190 (*abnA*)	ABN	GH43	5.5	−2.4	1.8	1.4	13.1	nd	AraR, GaaR	[34, 83]
	An01g10350 (*lacB*)	LAC	GH35	1.9	**−2.7**	1.3	3.5	12.9	AraR	AraR, GaaR	[15, 34, 38]
	An18g05940 (*galA*)	GAL	GH53	–	–	–	–	–	nd	AraR, GaaR, RhaR	[34, 84]
	An01g12150 (*lacA*)	LAC	GH35	**−8.9**	**−6.3**	1.4	6.4	13.6	AraR, XlnR	AraR, XlnR	[23, 34, 85]
	An08g10780 (*gbgA*)	BXL	GH43	−1.6	**−29.7**	2.8	11.7	5.2	AraR	AraR	[34, 39]
	An08g01710 (*abfC*)	ABF	GH51	1.8	**−8.7**	1.8	7.7	31.1	AraR	AraR, GaaR	[15, 34, 39]
	An15g02300 (*abfB*)	ABF	GH54	2.2	**−6.2**	1.3	18.8	159.4	AraR	AraR	[34, 46]
G-3	An01g01320	AGL	GH27	–	−**3.8**	–	–	–	AraR	nr	[38]
	An17g00300 (*xarB*)	BXL/ABF	GH3	**−2.8**	−1.9	1.2	1.2	1.0	XlnR	nr	[47]
	An01g04880 (*axlB*)	AXL	GH31	−2.2	1.0	1.8	1.8	1.2	nd	nr	[41]
	An16g02760 (*afcA*)	AFC	GH95	**−5.2**	2.2	−1.3	1.7	5.9	XlnR	nr	[38]
	An01g03340 (*xgeA*)	XG-EGL	GH12	−1.1	−2.1	–	–	–	nd	nr	[38]
	An04g09690 (*pmeB*)	PME	CE8	–	–	–	–	–	nd	AraR, GaaR	[15, 34, 39]
	An04g09700 (*xghA*)	XGH	GH28	–	**−4.6**	–	–	–	AraR	AraR, GaaR	[34, 86]
	An01g11520 (*pgaI*)	PGA	GH28	–	–	–	–	–	nd	GaaR	[15, 34, 50]
	An19g00270 (*pelD*)	PEL	PL1	–	–	–	–	–	nd	AraR, GaaR, RhaR	[15, 34, 72, 87]

^a^Genes with expression value of <50 in all studied *A. niger* N402 cultures are not included in the table

^b^Enzyme codes are provided in Additional file 2

^c^Fold-change between *A. niger* N402 and the regulatory mutants grown on their relevant carbon source. - = expression value <50 in both N402 and regulatory mutant strain. Negative fold-changes >2.5 were considered proof of regulator function and are depicted in bold

*na* no assigned or predicted function, *nd* not detected, *nr* not reported

**Table 10:** Significantly expressed *A. niger* N402 genes from cluster H in the expression profiling tree

				Up/Down-regulated^c^					Regulated by		
	Gene^a^	Enzyme^b^	CAZy family	Δ*xlnR*	Δ*araR*	Δ*rhaR*	Δ*galX*	Δ*amyR*	This study	Literature	Reference
H-1	An04g03170	BGL	GH1	–	–	–	2.3	3.3	nd	nr	[38]
	An14g01770	BGL	GH3	1.0	1.5	−1.2	1.0	1.5	nd	AmyR	[8, 38]
	An11g00200 (*bg1M)*	BGL	GH3	–	3.7	–	–	2.2	nd	nr	[38]
	An12g02550 (*faeC*)	FAE	CE1	–	–	–	–	–	nd	nr	[39]
	An12g10390 (*faeB*)	FAE	SF1^d^	–	2.4	**−2.8**	1.1	6.5	RhaR	AraR, GaaR, RhaR	[10, 34, 84]
H-2	An15g04570	LPMO	AA9	–	**−2.7**	1.3	1.3	–	AraR	nr	[38]
	An14g04200 (*rhgB*)	RHG	GH28	–	–	–	–	–	nd	nr	[88]
	An16g09090	na	GH3	−1.1	1.2	−1.1	1.2	2.0	nd	nr	[38]
	An18g03570 (*bglA*/*bgl1*)	BGL	GH3	**−13.1**	2.9	−2.4	1.5	101.3	XlnR	nr	[53]
	An16g02100	EGL	GH5	−1.4	−1.1	1.3	1.3	1.7	nd	nr	[38]
	An04g02700	AGL	GH36	–	–	1.2	1.6	–	nd	AmyR	[8, 38]
	An18g04100 (*gp43*)	EXG	GH5	–	**−5.7**	−1.5	6.0	1.8	AraR	nr	[38]
	An01g06120 (*gdbA*)	GDB	GH13	−1.2	1.0	−2.1	1.9	6.3	nd	nr	[41]
	An01g10930 (*agdB*)	AGD	GH31	2.2	3.4	1.0	1.9	1.3	nd	AmyR	[8, 41]
	An11g03120 (*xynD*)	BXL	GH43	−1.7	4.5	1.2	4.9	22.5	nd	nr	[38]
	An06g00170 (*aglA*)	AGL	GH27	–	**−4.2**	−1.6	6.5	–	AraR	AmyR	[8, 89, 90]
	An02g11150 (*aglB*)	AGL	GH27	−1.5	1.1	2.5	9.6	5.5	nd	XlnR	[23]
	An02g13240 (*agdC*)	AGD	GH13	1.0	1.1	−1.2	2.8	2.4	nd	nr	[41]
	An05g02410	GUS	GH2	–	1.7	1.9	4.5	3.9	nd	nr	[38]
	An14g04190 (*gbeA*)	GBE	GH13	−1.7	1.3	−1.6	2.9	6.1	nd	nr	[41]

^a^Genes with expression value of <50 in all studied *A. niger* N402 cultures are not included in the table

^b^Enzyme codes are provided in Additional file 2

^c^Fold-change between *A. niger* N402 and the regulatory mutants grown on their relevant carbon source. - = expression value <50 in both N402 and regulatory mutant strain. Negative fold-changes >2.5 were considered proof of regulator function and are depicted in bold

^d^Sub-family (SF) classification of fungal FAEs according to Dilokpimol et al. [49]

*na* no assigned or predicted function, *nd* not detected, *nr* not reported

**Table 11:** Significantly expressed *A. niger* N402 genes from clusters A and I in the expression profiling tree

				Up/Down-regulated^c^					Regulated by		
	Gene^a^	Enzyme^b^	CAZy family	Δ*xlnR*	Δ*araR*	Δ*rhaR*	Δ*galX*	Δ*amyR*	This study	Literature	Reference
A	An15g00320 (*sucB*)	SUC	GH32	1.5	−1.3	−2.1	–	1.7	nd	InuR	[9, 52]
	An15g07160 (*pelF*)	PEL	PL1	1.5	1.4	−2.1	−1.1	1.7	nd	GaaR	[15, 35, 72]
I	An08g05230	LPMO	AA9	–	–	**−2.9**	−1.2	1.3	RhaR	nr	[38]
	An14g02670	LPMO	AA9	−1.1	−1.7	−1.6	1.0	–	nd	XlnR	[21, 38]
	An03g05380	XG-EGL	GH12	–	–	–	–	–	nd	nr	[38]
	An10g00870 (*plyA*)	PLY	PL1	–	–	–	–	–	nd	nr	[91]
	An02g10550 (*abnC*)	ABN	GH43	**−27.8**	15.2	1.4	−1.8	49.2	XlnR	nr	[39]
	An15g03550	ABN	GH43	–	10.0	–	–	6.0	nd	nr	[38]
	An07g07630	BGL	GH3	–	2.9	–	–	3.1	nd	nr	[4]
	An11g06080	BGL	GH3	–	2.0	1.4	2.4	3.8	nd	nr	[4]

^a^Genes with expression value of <50 in all studied *A. niger* N402 cultures are not included in the table

^b^Enzyme codes are provided in Additional file 2

^c^Fold-change between *A. niger* N402 and the regulatory mutants grown on their relevant carbon source. - = expression value <50 in both N402 and regulatory mutant strain. Negative fold-changes >2.5 were considered proof of regulator function and are depicted in bold

*nd* not detected, *nr* not reported

### AraR and XlnR regulated genes involved in cellulose, xyloglucan, xylan and arabinan degradation cluster together based on their expression profile

The genes of cluster B were specifically induced on L-arabinose, D-xylose and/or polygalacturonic acid. This cluster can be divided into sub-clusters B-1 and B-2 that contain seven and ten significantly expressed genes, respectively (Additional file 1, Table 4). The highest expression for genes of sub-cluster B-1 was detected on L-arabinose, except for *axhA* that was also induced on L-arabinose, but was higher expressed on polygalacturonic acid. The *axhA* gene encodes an arabinoxylan arabinofuranohydrolase and is specific for arabinoxylan degradation [30]. The high expression of this and other genes of cluster B on polygalacturonic acid may be due to impurity of the substrate (Table 3). The expression of XlnR-regulated genes has been shown to decrease with increasing concentrations of D-xylose due to carbon catabolite repression [31]. Small traces of D-xylose and L-arabinose in the polygalacturonic acid substrate may therefore lead to higher expression of the xylanolytic, arabinanolytic and cellulolytic genes than on 25 mM of D-xylose or L-arabinose used in our study. One gene of sub-cluster B-1 has been characterized as an endoglucanase (*eglB*), which has activity towards cellulose [21]. The other significantly expressed genes of this cluster encode an arabinofuranosidase (*abfA*), two putative endoxylanases (*xlnC* and *xynA*), a putative α-galactosidase (*aglD*) and a putative β-endogalactanase (An03g01050) (Table 4).

The highest expression level of these genes was found on L-arabinose (Additional file 1). Regulation of two of these genes, *abfA* and *axhA*, is controlled by the transcriptional activator AraR [23] that is induced by L-arabitol, a metabolic product of L-arabinose [32]. Co-regulation of AraR-regulated arabinanolytic genes (i.e. *abfA*, *abfB* and *abnA*) has been suggested previously [22, 33] and *abfA* has been shown to be controlled by GaaR [15, 34]. A previous principal component analysis (PCA) clustering of the pectinolytic genes has been shown to result in one cluster which contained *abfA*, *abfB*, *abnA* and *lacA* [35], which matches a more resent hierarchical clustering of the expression of pectinolytic genes in wild type and *gaaR* deletion mutant strains resulting in a cluster containing *abfA, abfB, abfC, lacA, lacB* and An03g01620 [15]. However, in this study, the *abfB*, *abnA* and *lacA* genes were separated from *abfA*, which indicates that *abfA* has a distinct expression profile from the other genes. This is likely due to the large set of carbon sources that were tested in our study, which provides a more detailed view of the expression of these genes than has been published previously, and also reveals the complexity of the expression of plant-biomass related genes. It should be noted that in nature, fungi are confronted with mixtures of carbon sources, and therefore likely activate a combination of the gene sets we observed in response to pure substrates.

Similar expression profiles for the other genes in this sub-cluster (*eglB*, *xlnC*, *aglD*, *xynA* and An03g01050) suggest that they are also regulated by AraR. This is supported by the reduced expression of these genes in the Δ*araR* strain on L-arabinose compared to N402 (Table 4). The *axhA* and *xlnC* genes are also regulated by XlnR [21], which was confirmed in our analysis, as these genes were down-regulated in the Δ*xlnR* strain. In addition, *xlnC* has been reported to be ClrA-regulated [13]. Thus, our results indicate a broader role for AraR as some of the genes related to cellulose (*eglB*), galactomannan (*aglD*, *mndA*), pectin (*lacA*, *lacB*, *xghA*), xyloglucan (*axlA*) and xylan (*gbgA*, *xlnB*, *xlnC*, An01g01320) degradation were significantly down-regulated in the Δ*araR* strain.

The genes of sub-cluster B-2 were significantly down-regulated in the Δ*xlnR* strain (Table 4), thus suggesting that they are controlled by XlnR. Indeed, five of these genes (*axeA*, *xlnD*, *aguA*, *faeA* and *xlnB*) have previously been shown to be regulated by XlnR [21, 36, 37]. The highest expression for most genes of this sub-cluster was detected on D-xylose, except for *aguA* and An11g02100 that were higher expressed on polygalacturonic acid, and *axlA* and An16g00540 that were higher expressed on L-arabinose (Additional file 1). High expression of *axlA* on D-xylose has previously been reported [9, 37]. This gene encodes a putative α-xylosidase that is suggested to have a role in xyloglucan degradation [38]. An16g00540 encodes an α-L-fucosidase, which also has a putative role in xyloglucan hydrolysis, indicating co-regulation of some of the genes involved in this process. An11g02100 and An08g01760 encode the cellulolytic enzymes β-glucosidase and cellobiohydrolase, respectively. This is in line with the previous finding that XlnR is a regulator of xylanolytic, xyloglucanolytic and cellulolytic genes [21]. The co-regulation of AraR- and XlnR-regulated genes in cluster B that are involved in cellulose, xyloglucan, xylan and arabinan degradation supports combined action of regulators. Co-regulation of these genes is an efficient strategy for polysaccharide degradation, since L-arabinose, D-xylose and D-glucose often co-occur in plant cell wall polysaccharides.

### Expression of pectinolytic genes involved in degradation of the pectin main chains were clustered

Cluster C contains 28 significantly upregulated genes of which most are pectin backbone hydrolyzing genes, mainly from CAZy families GH28 (several types of pectin hydrolases) and GH78 (α-rhamnosidases) (Table 5). It can be divided into the sub-clusters C-1, C-2 and C-3 (Additional file 1). Sub-cluster C-3 contains 12 significantly expressed genes, of which 10 have been shown to be regulated by RhaR and are specifically induced on L-rhamnose [10]. The other two genes of this cluster, *agdF* and An01g14600, were also specifically induced on L-rhamnose and down-regulated in the Δ*rhaR* strain suggesting that they are also under control of this regulator (Table 5). However, our results suggest a broader role for RhaR, since in addition to its target genes of cluster C, some other genes were identified that were down-regulated in the Δ*rhaR* strain, such as *aguA*, *aglC* and *mndA*.

Notably, the *agdF* gene has previously been assigned to encode a putative enzyme of the starch degrading GH31 family [38]. Our data does not support a function in starch degradation as, in addition to induction on L-rhamnose, this gene was significantly up-regulated in the Δ*amyR* strain (Table 5), while the opposite would be expected for a starch-related gene. The expression profile of An01g14600, which encodes a putative enzyme of the GH11 endoxylanase family, is unexpected as no link between this family and rhamnogalacturonan degradation has been described. Therefore, our data suggests the involvement of *agdF* and An01g14600 in rhamnogalacturonan degradation, although their enzymatic function is unclear at this point. A high expression level on L-rhamnose has been previously reported for *rgaeB*, *rgxA*, *rgxB*, *urhgA* and *rglB* [39, 40]. In our analysis, *rgaeB* appears to have a slightly different expression profile from the other genes of sub-cluster C-3 as it is located in a separate branch of the hierarchal cluster (Additional file 1). The inclusion of the L-rhamnose and D-galacturonic acid mixture data enabled us to evaluate the co-operation of these two sugars as inducers by comparing them to the individual sugar cultivations. Interestingly, despite the dominant role for galacturonic acid and GaaR in regulation of pectinolytic genes [16, 34], the mixture of L-rhamnose and D-galacturonic acid clusters more closely with L-rhamnose than with D-galacturonic acid in our analysis. This may indicate that the induction by L-rhamnose is more discriminative than the induction by D-galacturonic acid in distinguishing genes by expression pattern.

Sub-cluster C-1 contains three significantly expressed genes, two of which are regulated by RhaR on L-rhamnose and by GaaR: *lacC* and *rgxC* (Table 5) [10, 15]. The *lacC* and *rgxC* genes were previously reported to be expressed on D-galacturonic acid, polygalacturonic acid and L-rhamnose, in contrast to the genes of sub-cluster C-3 that were specifically induced on L-rhamnose [39]. High expression of *lacC* and *rgxC* on galactan could be due to the small traces of D-galacturonic acid and L-rhamnose in the substrate (Table 3). The *lacC* has also been reported to be under control of AraR [34] and AmyR [8], but it was not observed to be down-regulated in the Δ*amyR* strain in our study (Table 5). The third gene of the sub-cluster C-1, *amyD*, has been classified as an α-amylase [38], but its expression was not detected on D-maltose in *A. niger* N402 [41]. In our study, the gene was expressed on D-galacturonic acid, polygalacturonic acid and the mixture of D-galacturonic acid and L-rhamnose (Additional file 1). A role for *amyD* in starch degradation is therefore doubtful.

The pectinolytic genes in sub-cluster C-2 are involved in the degradation of homogalacturonan (Table 5). These genes are not regulated by RhaR but were induced on D-galacturonic acid and polygalacturonic acid in this study (Additional file 1) and most of them are under control of GaaR [15, 34]. The significantly expressed genes of sub-cluster C-2 include four exopolygalacturonases (*pgxA*, *pgxB*, *pgxC* and *pgaX*), a pectin methyl esterase (*pmeA*), a pectin lyase (*pelA*), and rhamnogalacturonan lyase (*rglA*) (Table 5), all of which have been shown to be GaaR-regulated [15]. In addition, regulation by AraR has been reported for *pgxC*, and by AraR and RhaR for *pmeA* [34]. Also, gene An02g00140, which encodes a putative β-xylosidase, showed significant expression (Table 5). The expression profiles of *pelA*, *pmeA* and *pgaX* genes were previously shown to cluster and these genes were suggested to play a major role in the initial degradation of pectin [35]. This is also supported by the results reported from sugar beet pectin [15]. In line with our results, strong induction on D-galacturonic acid and polygalacturonic acid has been reported for *pgxB*, *pgxC* and *pgaX*, while lower expression has been observed for *pgxA* on these substrates [15, 40]. The *pelA* gene was well expressed on all tested substrates, but its highest expression was detected on polygalacturonic acid (Additional file 1). In agreement with the previous studies [15, 39], the *rglA* gene was expressed on D-galacturonic acid, polygalacturonic acid and galactan, but not on L-rhamnose. The GaaR-regulated *pmeA* gene [15] was slightly induced on D-galacturonic acid and polygalacturonic acid in our study and that of de Vries et al. [35]. In contrast to the results of Kowalczyk et al. [34], the regulation of *pmeA* by AraR or RhaR was not detected. The function of five out of eight putative α-rhamnosidase encoding genes (i.e. An01g06620, An12g05700, An07g00240, An10g00290 and An04g09070) in sub-cluster C-2 is supported by our analysis as they are specifically induced on L-rhamnose and are under control of RhaR [10, 38]. In addition, An12g05700 is controlled by GaaR and RhaR, and An18g04800 by GaaR [34].

The pectinolytic genes found in cluster C were expressed on L-rhamnose, D-galacturonic acid and/or polygalacturonic acid, suggesting that these genes encode initial pectin degrading enzymes. Pectinolytic genes that showed no significant, or constitutive expression, may be induced on pectin-related substrates after longer incubation times. Expression of *plyA*, *pgaII*, *pgaB*, *pgaD*, *pgaE*, *pelB*, *pelC* and *pelF* was low or not significant on all substrates in our study. However, expression of these genes on D-galacturonic acid, polygalacturonic acid and sugar beet pectin has been reported to increase in time [35] and *pgaB*, *pgaE* and *pelF* have been shown to be regulated by GaaR [15, 34].

### Constitutively expressed genes clustered with genes involved in starch degradation

In cluster D, sub-cluster D-1 contains nine significantly expressed genes encoding enzymes from different GH families, while in sub-cluster D-2 six genes are present that mainly encode enzymes from GH families assigned to starch degradation (GH13, 15 and 31) (Table 6). The genes of sub-cluster D-1 were not down-regulated in any of the tested regulatory mutant strains, indicating that they are not regulated by these transcriptional activators (Table 6). They show a relatively distant separation from each other, and most showed low, but similar expression levels on all substrates (Additional file 1) indicating that the genes in sub-cluster D-1 are likely constitutively expressed. Indeed, the *abnB* gene, present in sub-cluster D-1, was previously reported to be constitutively expressed on D-fructose, D-xylose, sorbitol, L-rhamnose, D-galacturonic acid, polygalacturonic acid and sugar beet pectin [39].

The sub-cluster D-2 contains genes that are involved in starch degradation and are down-regulated in the Δ*amyR* strain. Two *glaA* and *agdA* genes, encoding a glucoamylase and an α-glucosidase, respectively [38, 41], showed high expression on all substrates, while the highest expression levels were detected in N402 on maltose (Additional file 1), in line with the previous study [41]. Gene *aamA*, which encodes an acid α-amylase [42], has also been reported to be highly expressed on maltose [41], but was expressed at a much lower level in our study. For this gene, significant expression was also detected on L-arabinose, polygalacturonic acid and sugar beet pulp (Additional file 1). The similar expression patterns and the down-regulation of *glaA*, *agdA* and *aamA* genes in the Δ*amyR* strain (Table 6) indicates their co-regulation by AmyR, as has been suggested by Yuan et al. [41]. All three genes were up-regulated in the Δ*galX* mutant on D-galactose to a higher level than the expression on maltose in N402 (Additional file 1). The α-amylase gene *amyC* was also most highly expressed on D-galactose in the Δ*galX* mutant. Like *glaA*, *agdA* and *aamA*, expression of this gene was reported to be reduced in the Δ*amyR* strain [41]. However, the expression profile of *amyC* in our study differs from the other three amylolytic genes, because a similar expression level of this gene was found on D-maltose, L-rhamnose and guar gum, making its induction on D-maltose less specific (Additional file 1). In a previous study, expression of *amyC* was similar on D-xylose and D-maltose after 2 h of incubation, but the gene was not expressed after 8 h on xylose, while its expression on maltose was still detected [41].

Low expression for *agtB* encoding a putative 4-α-glucanotransferase was detected on all substrates, with only significant expression levels and down-regulation in the Δ*amyR* strain (Additional file 1, Table 6). This data is in contrast with a previous study [41], where expression was only detected after 8 h on D-maltose and *agtB* was reported to be AmyR independent. Co-expression of *agtB* and *agsC*, encoding a putative α-glucan synthase, has previously been observed [41]. Even though *agsC* was not significantly expressed in our study (Additional file 2), it did cluster with *agtB* in our initial correlation analysis (Additional files 1 and 2).

An03g05530 is also found in sub-cluster D-2, even though its highest expression level was detected on L-rhamnose and D-galacturonic acid. However, this gene is significantly down-regulated in the Δ*amyR* strain, which may explain its presence in sub-cluster D-2.

### InuR-regulated inulinolytic genes were co-expressed on sucrose and inulin

Cluster E contains eight significantly expressed genes that have relatively distant positions in the expression profile tree (Additional file 1). Sub-cluster E-1 consists of only *eglD* and *pelB* encoding a putative LPMO and a pectin lyase, respectively (Table 7), that showed a low overall expression. While this is in contrast to the reported lack of expression for *pelB* in *A. niger* cultures on sugar beet pectin, galacturonic acid, rhamnose and xylose [39], the low expression we observed may indicate that expression levels of *pelB* are always around the detection cut-off. Sub-cluster E-2 contains six genes that were expressed on guar gum, inulin, sugar beet pulp and/or sucrose (Table 7, Additional file 1). High expression on inulin and to a lesser extent on guar gum was observed for a putative exo-inulinase encoding gene *inuE*, which clustered with an endo-inulinase encoding *inuA,* but expression levels of the latter gene were much lower. In addition to *inuE* and *inuA*, sub-cluster E-2 contains the extracellular inulinolytic gene *sucA*. These genes were all regulated by InuR, and co-regulation and expression on sucrose and inulin was previously reported for these genes [43]. The more distant position of *sucA* in the expression profile tree can be explained by its relative expression levels on sucrose, inulin and sugar beet pulp, the latter resulting in the highest expression for *sucA*. An08g01100 and to a lesser extent An07g09760 were specifically induced on guar gum, but are located close to *inuE* and *inuA* in the expression profile tree (Additional file 1). The correlation analysis also demonstrated which substrates are most similar when the expression of all the tested genes was taken into account. Guar gum was most closely related to inulin, sucrose and sugar beet pulp. The sugar beet pulp used in this study contains significant amounts of sucrose (data not shown), which explains the clustering of this substrate with sucrose and inulin. Our results suggest that guar gum may also contain some traces of sucrose, even though this was not reported by the supplier.

Other inulinolytic genes described for *A. niger*, i.e. *sucB*, *sucC* and *inuQ*, were not present in cluster E. Absence of expression of the intracellular invertase encoding *sucC* gene, and *inuQ*, which was described to be a pseudogene, confirmed a previous study [44]. The other intracellular invertase encoding gene, *sucB*, was reported to have an overall low expression on other substrates than sucrose and inulin [44], which was also confirmed by our study.

Only five significantly expressed genes are positioned in cluster F (Additional file 1, Table 8), with only one gene, An09g05350, in sub-cluster F-2. It was expressed on D-glucose, D-fructose, D-maltose and rhamnogalacturonan. The four genes that form sub-cluster F-1 differ in their expression profile, and therefore the reason for the clustering of these genes may be that they did not fit into any of the other clusters. It should be noted that the genes of cluster F are distantly separated from each other within the expression profiling tree (Additional file 1). A putative α-glucan synthase encoding gene (*agsE*) showed high expression levels on all substrates in N402, which confirms a previous study [41]. However, expression of this gene was strongly reduced in the Δ*amyR* strain (Table 8), which was not observed in the study of Yuan et al. [41]. The opposite was found for α-galactosidase encoding *aglC* that has been reported to be under control of AmyR [41], while our study only detected significant down-regulation in the Δ*rhaR* strain. Expression of endoarabinanase encoding *abnD* was previously reported to be constitutive [39], but more recently it was shown to be GaaR-dependent on D-galacturonic acid and GaaR and RhaR-dependent on sugar beet pectin [15, 34]. However, we only detected significant expression levels of *abnD* on D-maltose in N402 and down-regulation in the Δ*amyR* strain, suggesting control by this regulator.

### Genes related to degradation of pectin side chains cluster separately from those acting on the pectin main chain

Most of the significantly expressed genes of cluster G (Table 9) were highly expressed on D-galacturonic acid and polygalacturonic acid (Additional file 1). The difference between these genes and D-galacturonic and polygalacturonic acid induced genes of cluster C is that the cluster G genes are less specifically induced on D-galacturonic acid and polygalacturonic acid, as they also show high expression levels on other carbon sources. Cluster G, the largest cluster detected with 23 genes, can be divided into the sub-clusters G-1, G-2, and G-3 (Additional file 1).

Expression of some of the genes in cluster G has been previously analyzed on D-fructose, L-rhamnose, D-xylose, sorbitol, D-galacturonic acid, polygalacturonic acid and sugar beet pectin [15, 39, 40]. Specific induction has been observed for *pmeB*, *xghA*, *pgaI*, *abfB*, *abfC*, *lacA*, *lacB*, *galA* and *abnA* on D-galacturonic acid, polygalacturonic acid and sugar beet pectin [15, 45], and all these genes have been shown to be GaaR-controlled, except *lacA* and *abfB* [15, 34]. Furthermore, the *abfB* and *abfC* genes were also highly expressed on D-xylose [39, 46]. In our study, induction of these genes on D-galacturonic acid and polygalacturonic acid was also observed. In addition, *abfB*, *abfC*, *lacA*, *lacB*, *galA* and *abnA*, all members of sub-cluster G-2, were highly expressed on galactan (Additional file 1). Co-regulation of *abfB*, *abnA* and *galA* was suggested previously [33, 35], but in our study only *abnA* and *galA* fall in the same cluster, while the expression profile of *abfA* is different.

High expression for most of the sub-cluster G-2 genes, except *lacA* and *galA*, was observed on arabinan, while high expression on L-arabinose was observed for *abfB*, *abfC* and *lacA*, all of which were down-regulated in the ∆*araR* strain. The genes of this sub-cluster all encode enzymes that could be involved in the degradation of the pectinolytic side chains, suggesting a strong link between function and expression.

High expression levels of the genes of sub-cluster G-1 were detected on polygalacturonic acid, but to a much lower extent than for the sub-cluster G-2 genes. The highest expression for three genes of sub-cluster G-1, *mndA*, *mndB* and *bgl4*, was found on mannan. The *mndA* gene encodes a β-mannosidase [44], involved in mannan degradation, while *mndB* and *bgl4* encode a putative β-mannosidase and β-glucosidase, respectively. Their co-expression with *mndA* supports these functions as both activities are needed for complete degradation of galactoglucomannan. However, these genes were not inducted by mannose. This is in line with the ManR/ClrB regulator from *A. oryzae* induced by mannobiose, but not by mannose [11, 12]. The highest expression for the other genes of this sub-cluster, *agdE*, An17g00520 and An02g07590, was detected on polygalacturonic acid.

The highest expression levels of all the genes of sub-cluster G-3 were found on polygalacturonic acid. The GaaR, AraR and RhaR-regulated *pelD* gene [15, 34] was specifically induced on polygalacturonic acid, in contrast to a previous study where this gene was reported to be non-expressed [39]. Expression of *pgaI*, which is under control of GaaR [15], and *pmeB* and *xghA*, which are under control of GaaR and AraR [15, 34], has previously been reported on D-galacturonic acid and polygalacturonic acid [39], which was confirmed in our study. Three genes of sub-cluster G-3, *xarB*, *axlB* and *afcA*, which encode a putative bi-functional xylosidase/arabinofuranosidase [47], an α-glucosidase and an α-fucosidase, respectively, were down-regulated in the Δ*xlnR* strain, suggesting control by XlnR. One gene of this sub-cluster, *xghA*, was down-regulated in the Δ*araR* strain, suggesting regulation by AraR in line with Kowalczyk et al. [34]. As mentioned earlier, the polygalacturonic acid specific induction of arabinanolytic and xylanolytic genes may be due to impurity of the substrate with small traces of D-xylose and L-arabinose.

### Cluster H contains a diverse set of genes that are expressed on a broad range of substrates

Six and 15 significantly expressed genes form sub-cluster H-1 and H-2, respectively (Additional file 1, Table 10). The *faeB* gene was expressed at a basal level on L-rhamnose, D-xylose, sorbitol, D-fructose D-galacturonic acid, polygalacturonic acid and sugar beet pectin, while *faeC*, which is also found in this sub-cluster, was not expressed on these substrates [39]. The genes of sub-cluster H-1 were all specifically induced on ferulic acid. While induction of *faeB* on ferulic acid has previously been reported [48], the *faeC* was also induced on this substrate, suggesting co-regulation of these two feruloyl esterase encoding genes, which was confirmed by a recent study [49]. Interestingly, the other genes of the sub-cluster H-1 specifically induced on ferulic acid encode putative β-glucosidases (An04g03170, An14g01770 and *bgm1*) and a putative LPMO (An15g04570).

The genes of sub-cluster H-2 were expressed at a constant level on most carbon sources tested, but showed low expression on D-glucose, D-fructose, sucrose and sugar beet pulp (Additional file 1). As mentioned before, the sugar beet pulp used in this analysis contains sucrose (data not shown). These genes may therefore be under strong carbon catabolite repression. Binding sites for CreA have been found in the promoter regions of all these genes [38], and low overall expression of a putative α-glucosidase encoding *agdC* has previously been described [41]. Another α-glucosidase encoding gene, *agdB*, has been reported to be strongly induced on D-maltose and down-regulated in the Δ*amyR* strain [41]. Our study, however, revealed that this gene was highly expressed on most carbon sources tested and no down-regulation in the Δ*amyR* strain was observed (Table 10).

### Two clusters of putatively not co-expressed genes were detected

In clusters A and I, only a small number of genes (two and eight, respectively) were significantly expressed (Additional file 1, Table 11). Furthermore, the genes in clusters A and F share no specific trends in their expression profiles and are relatively distantly separated from each other within the expression profiling tree (Additional file 1), and are probably not co-expressed.

In cluster A, the significantly expressed genes, *sucB* and *pelF*, encode enzymes from CAZy families GH32 and PL1, respectively (Table 11, Additional file 1). The overall expression of these genes was very low on all substrates. Furthermore, the genes were not significantly down- or up-regulated in the studied regulatory mutant strains, indicating that these genes are not regulated by any of these transcriptional activators. The low overall expression of *pelF*, a gene encoding a putative pectin lyase, has been reported previously [39] and it has been shown to be regulated by GaaR [15]. Notably, *pelF* did not cluster with any of the other pectinolytic genes in our data. In contrast, *pelF* clustered distantly with the other pectinolytic genes in a previous study [35], which, however, included a smaller set of genes and a more focused set of growth conditions that may explain the differences with our study. In addition, the previous hierarchical clustering suggested induction of *pelF* during starvation or derepressed conditions [15]. Gene *sucB* encodes an intracellular invertase with transfructosylation activity [50, 51]. Its expression profile was distinct from other inulinolytic genes (Additional file 1). The *sucB* gene has been reported to be under control of the inulinolytic regulator InuR [52] and to be constitutively expressed at low level [43]. In our study, significant expression of *sucB* was found on inulin, which supports regulation by InuR. In addition, *sucB* expression was observed on D-maltose in the Δ*amyR* strain (Additional file 1). This suggests interaction between AmyR and InuR, similarly as was described for XlnR and AraR in *A. niger* [22, 53].

All genes in cluster I were expressed at low level on D-maltose and sugar beet pulp in the N402 strain. Some of these genes (i.e. An15g03550, *abnC*, An07g07630 and An11g06080) were up-regulated in the Δ*amyR* strain. The highest expressed gene of this cluster was a putative endoarabinanase encoding *abnC*, which was highly expressed on all the tested substrates except D-maltose and sugar beet pulp (Additional file 1). Expression levels of this gene have previously been reported to be elevated after 24 h on D-fructose, L-rhamnose, sorbitol, D-xylose and D-galacturonic acid [39]. The *abnC* gene was significantly down-regulated in the Δ*xlnR* strain on D-xylose, which indicates that this gene is regulated by XlnR (Table 11). The *abnC* gene and An15g03550, both encoding putative endoarabinanases from family GH43, were highly expressed on galactan, while An15g03550 was also highly expressed on mannan. The highest expression levels of An08g05230 and An14g02670 encoding putative LPMOs from family GH61, An03g05380 encoding putative xyloglucan-active endoglucanase and *plyA* encoding putative pectate lyase were detected on arabinan (Additional file 1).

### Upregulation of genes in regulatory mutants suggests interaction between the different regulatory systems

While the down-regulation of gene expression in *A. niger* regulatory mutants compared to the wild type strain can be taken as evidence of control by this regulator, we surprisingly also found a significant number of genes for which the expression in a regulatory mutant was higher than in the wild type. While in most cases this was a moderate increase (less than 3-fold,), for 46 genes the difference was higher and 13 of these had fold-changes >10. The largest set of strongly upregulated genes was observed in the *amyR* mutant on maltose. Interestingly, this seems to especially affect L-arabinose related genes as the fold-change for *abfA*, *abfB*, *abfC*, *abnA*, *abnC* and An159g3550 (putative ABN) was 38, 160, 31, 13, 49 and 6, respectively. In addition, *bglA* was also 100-fold upregulated. Antagonistic interactions between regulators have been observed before, in particular for the two pentose-related regulators XlnR and AraR [54]. However, more recently, this was also observed for three pectinolytic regulators, GaaR, AraR and RhaR [34], suggesting that this is more common phenomenon has been so far considered. The nature of the antagonistic interaction and whether this is a direct or indirect is not clear at this point and requires further study.

## Conclusions

**Fig. 1:**
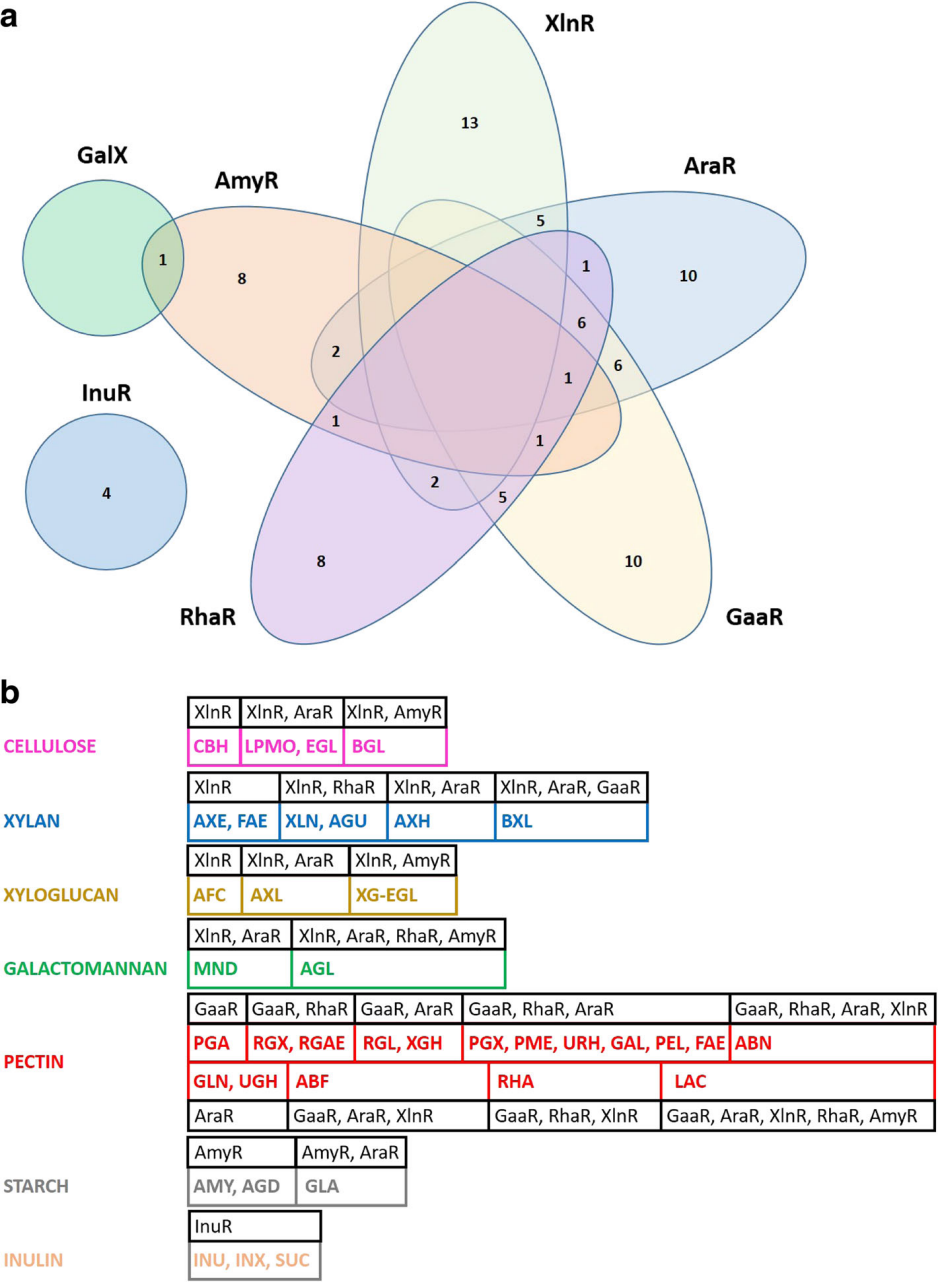
Global analysis of the expression profiles of CAZy genes related to plant polysaccharide degradation. **a** Number of genes under control of one or more regulators are indicated in a VENN diagram. **b** Comparison of the influence of regulators on enzyme activities linked to the polysaccharide they act on. Regulatory effects on individual genes encoding the same enzyme activity were combined in the boxes

This study aimed to reveal co-expression patterns of plant biomass polysaccharide degradation related genes from *A. niger*, using a more global approach than is usually performed by including a wide range of carbon sources, as well as five regulatory mutants, thus generating an unprecedented view of this system. The broader range of substrates revealed the highly complex expression patterns of these CAZy genes, and demonstrated that the focused analyses of the transcriptional regulators involved in this process that have been identified so far only revealed initial indications of the overall regulatory system. In fact, many of the genes tested in this study were shown to be under control of more than one regulator (Fig. 1a). Interestingly, the role of the regulators appears to be less linked to a specific polysaccharide when the genes encoding a certain enzyme activity and the regulators that act on them were combined (Fig. 1b). This could imply that the role of the enzymes may in fact be broader than currently assumed. E.g. the role of BXL in removing xylose from xylogalacturonan could explain the influence of GaaR on the expression of some BXL-encoding genes.

Previous studies in *T. reesei* and *N. crassa* also addressed induction of CAZy genes under different conditions and in some cases by using deletion mutants of plant biomass related regulators [55–58]. However, these studies, similar to previous studies in *Aspergillus*, used a limited number of carbon sources and did not compare different regulatory mutants. It can therefore be expected that also in these studies the complexity of the regulatory network driving plant biomass degradation is underestimated. More detailed studies in *A. niger* as well as other fungi are needed to fully grasp the organization of the regulatory network and reveal the differences between fungal species.

## Methods

### Strains, media and culture conditions

The *A. niger* strains used in this study are listed in Table 2. Strains were grown at 30 °C on minimal medium (MM) or complete medium (CM) [51] either or not containing 1.5% agar. Liquid cultures were grown on a rotary shaker at 250 rpm. Pre-cultures for RNA isolation were grown for 16 h in 1 L Erlenmeyer flasks that contained 250 ml CM supplemented with 2% D-fructose. Mycelium was washed with MM and 1 g (wet weight) aliquots were transferred for 2 h to 250 ml Erlenmeyer flasks containing 50 ml MM supplemented with 25 mM mono- or disaccharide or ferulic acid, or mixture of 25 mM L-rhamnose and 25 mM D-galacturonic acid, or 1% polysaccharide or complex plant biomass (Table 3). The only exceptions were D-maltose cultures of N402 and ∆*amyR* strains that were incubated for 4 h and for which 1% maltose was used. These data originate from a different study [8], but were included to help with the grouping of the genes and assess the AmyR effect. Mycelium was harvested by vacuum filtration, dried between towels and frozen in liquid nitrogen. While N402 liquid cultures were performed on all carbon sources listed in Table 3 as well as on the mixture of L-rhamnose and D-galacturonic acid, the regulatory mutant strains Δ*xlnR*, Δ*araR*, Δ*amyR*, Δ*rhaR* and Δ*galX* were grown on D-xylose, L-arabinose, maltose, L-rhamnose and D-galactose, respectively, and L-rhamnose and D-galacturonic acid. All cultures were performed as biological duplicates.

### Microarray processing

RNA isolation and microarray hybridization were performed as described previously [59]. In brief, RNA for microarray analysis was extracted using TRIzol reagent (Invitrogen) and purified using TRIzol® Plus RNA Purification Kit (Sigma-Aldrich) according to the instructions of the manufacturer. The concentration of RNA was calculated from the absorbance at 260 nm in a spectrophotometer (Biochrom Libra S22). The quality of the RNA was analyzed with an Agilent 2100 Bioanalyzer using a RNA6000 LabChip kit (Agilent Technology). Microarray hybridization using the Affymetrix GeneChips *A. niger* Genome Array was performed at GenomeScan (Leiden, The Netherlands).

### Transcriptome analysis

Microarray data was analyzed using the Bioconductor tool package version 2.8 (http://www.bioconductor.org/) together with house-made Perl (version .5.0) and Python (version 3.0) scripts. Probe intensities were normalized for background by the robust multi-array average (RMA) method [60] using the R statistical language and environment [61]. This method makes use of only perfect match (PM) probes.

Normalization was processed by the quantiles algorithm. The median polish summary method [62] was used to calculate the gene expression values. Further statistical analyses were performed with the CyberT tool package using multiple testing (http://cybert.ics.uci.edu/). BayesAnova and paired BayesT-test tests were performed on each gene through pairing carbon sources, PPDE (Posterior Probability of Differential Expression) analysis and multiple hypothesis testing correction are performed on the *p*-values [63]. Adjusted cut off value of *p* < 0.05 was used to determine the statistical significance of gene expression difference. Reproducibility of the replicates was verified by PCA analysis (Additional file 4). Genome scale PCA analysis was performed with the gene expression values of the different samples. The PCA was generated using R (v3.40) statistical language and environment, the PCA function from FactoMineR package (v1.35) and plotted using ggplot2 package (v 2.2.1). Replicates are plotted using the same color. Due to the large amount of data, the calculation of the matrix was not possible.

### Gene expression clustering, visualization and annotation

Hierarchical clusters were made using complete linkage with the normalized expression data from selected CAZyme encoding genes by calculating the Pearson correlation distances [64]. Clusters were set manually based on the branch-length differences of the gene-tree. The genes were selected based on the annotation of the CAZy families and their (putative) role in plant biomass degradation. Clusters and expression correlation profiles were visualized by Genesis [65]. Genes with an expression value <50 were colored dark blue, the ones >1000 were colored red and the values ≥50 and ≤1000 were colored by a gradient of these 2 colors.

Gene functional annotations were based on previous study [1]. When the data of this study suggested a different function, this was verified by performing phylogenetic analysis of the CAZy family this gene belongs to. The phylogeny analysis was performed using all the *A. niger* genes of the corresponding family together with all functionally characterized fungal members of that family, which allowed us to verify to which activity this gene clustered.

## Additional files

Additional file 1: Expression profiling tree containing 168 *A. niger* genes encoding putative CAZymes (www.cazy.org). Clusters A-I can be distinguished. (PDF 1780 kb)

Additional file 2: Expression of selected CAZy genes. (XLSX 215 kb)

Additional file 3: Significantly and not significantly expressed genes encoding CAZymes in *A. niger* CBS513.88 in this study. (XLSX 19 kb)

Additional file 4: PCA analysis of the gene expression values of the biological duplicate samples revealing the reproducibility of the duplicates. (PDF 90 kb)

## Abbreviations

AA: Auxiliary activity
CAZy: Carbohydrate-active enzyme
CE: Carbohydrate esterase
CM: Complete medium
GH: Glycoside hydrolase
MM: Minimal medium
PL: Polysaccharide lyase
RMA: Robust multi-array average
