# Supplementary figures and images for: Expression-based clustering of CAZyme-encoding genes of Aspergillus niger
Source: BMC Genomics. 2017 Nov 23;18:900. doi: 10.1186/s12864-017-4164-x (PMC5701360; doi:10.1186/s12864-017-4164-x)

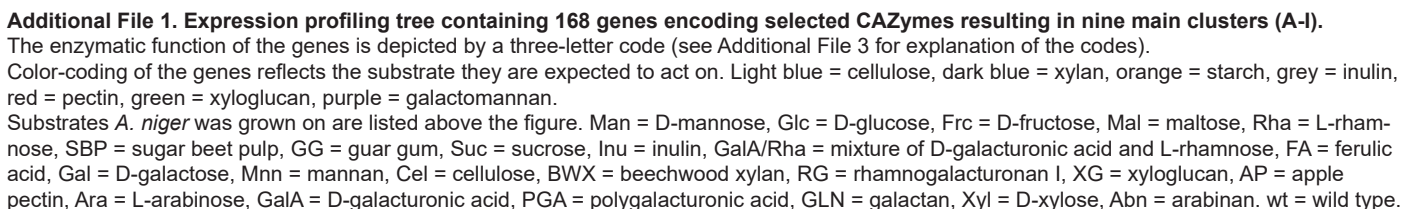

Supplement: Supplementary file 1 — Expression profiling tree containing 168 A. niger genes encoding putative CAZymes (www.cazy.org). Clusters A-I can be distinguished. (PDF 1780 kb) [file 12864_2017_4164_MOESM1_ESM.pdf]
