# Supplementary material for: Expression-based clustering of CAZyme-encoding genes of Aspergillus niger
Source: BMC Genomics. 2017 Nov 23;18:900. doi: 10.1186/s12864-017-4164-x (PMC5701360; doi:10.1186/s12864-017-4164-x)
Supplement: Supplementary file 4 — PCA analysis of the gene expression values of the biological duplicate samples revealing the reproducibility of the duplicates. (PDF 90 kb) [file 12864_2017_4164_MOESM4_ESM.pdf]

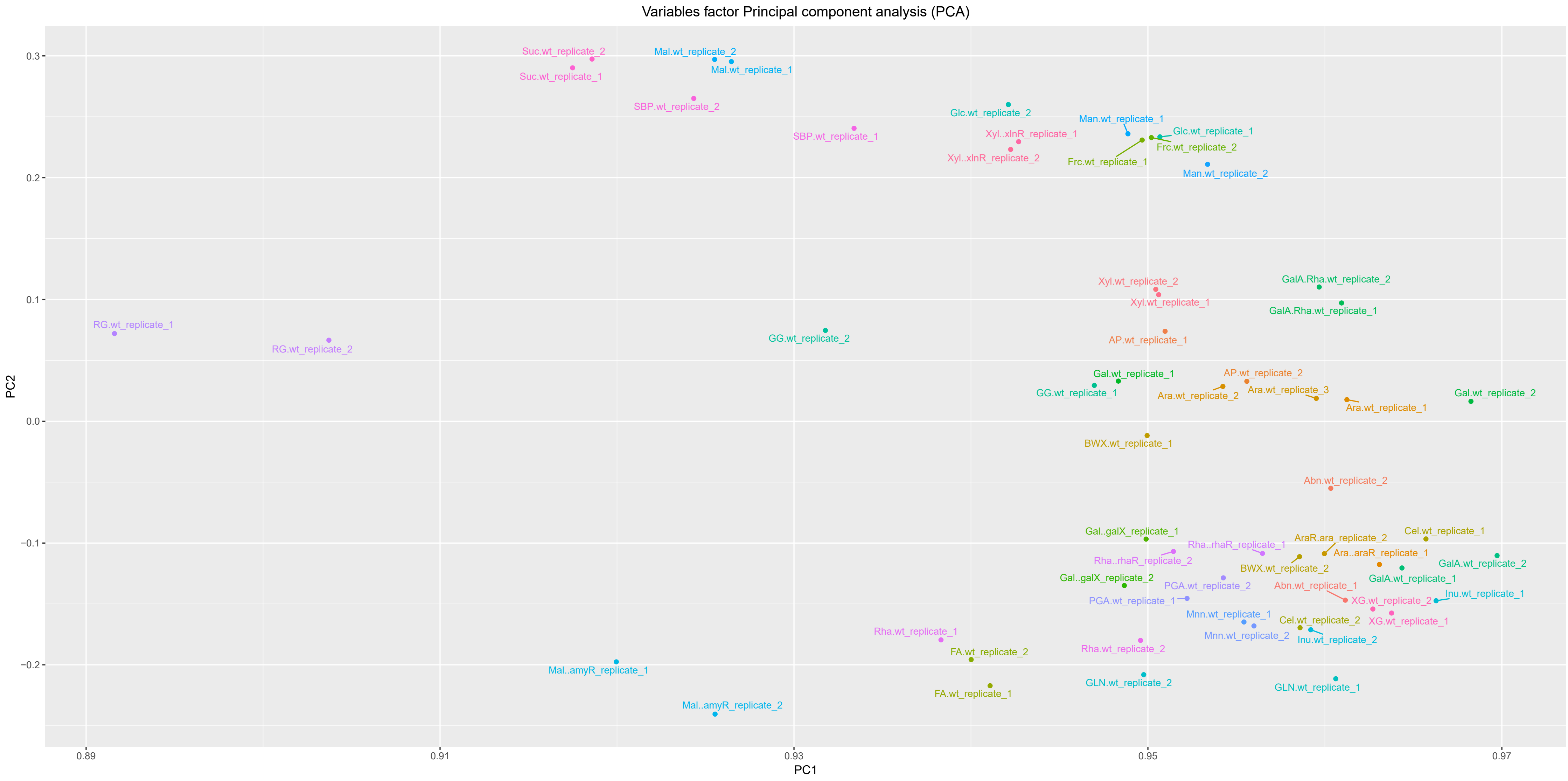

Additional File 4. PCA analysis was performed of the gene expression values of the biological duplicate samples revealing the reproducibility of the duplicates.
